# Primary care management of stroke in people with dementia: Linked registry and general practice data

**DOI:** 10.1111/ajag.70064

**Published:** 2025-07-11

**Authors:** Muideen T. Olaiya, Joosup Kim, Christopher Pearce, Dominique A. Cadilhac, Nadine E. Andrew, Lauren Sanders, Amanda G. Thrift, Mark R. Nelson, Seana Gall, Monique F. Kilkenny

**Affiliations:** ^1^ Stroke and Ageing Research, School of Clinical Sciences at Monash Health Monash University Clayton Victoria Australia; ^2^ Stroke Theme, Florey Institute of Neuroscience and Mental Health The University of Melbourne Heidelberg Victoria Australia; ^3^ Outcome Health Blackburn Victoria Australia; ^4^ Peninsula Clinical School, Central Clinical School Monash University Frankston Victoria Australia; ^5^ National Centre for Healthy Ageing Frankston Victoria Australia; ^6^ St Vincent's Hospital Melbourne Victoria Australia; ^7^ Department of Medicine, Melbourne Medical School University of Melbourne Melbourne Victoria Australia; ^8^ Menzies Institute for Medical Research University of Tasmania Hobart Tasmania Australia

**Keywords:** cardiometabolic risk factors, dementia, patient outcome assessment, primary health care

## Abstract

**Objectives:**

To evaluate whether risk factor management in Australian general practices for secondary prevention of stroke differs by dementia status.

**Methods:**

A retrospective study of adults with acute stroke or transient ischaemic attack (TIA) from 2014 to 2018, using de‐identified linked data (2014–2020) from the Australian Stroke Clinical Registry and three Primary Health Networks in Victoria. Eligibility included being discharged home or to inpatient rehabilitation, and having two or more encounters with general practice during the chronic phase (7–18 months) postindex stroke/TIA. We evaluated the assessment of cardiometabolic risk factors (blood pressure, serum lipids, blood glucose and urinary protein), prescription of prevention medications and attainment of risk factor targets, within 7–18 months postindex stroke/TIA. Regression models were used to determine any differences in risk factor management.

**Results:**

Among 3376 eligible survivors of stroke/TIA (median age 73.9 years, 22% TIA), 140 (4%) had evidence of a dementia diagnosis. In multivariable analyses, dementia was associated with fewer risk factors being assessed (incidence rate ratio [IRR] .86, 95% confidence interval [95% CI] CI .76–.98) or medication classes being prescribed (IRR .88, 95% CI .78–.98). No significant difference was observed in the attainment of risk factor targets.

**Conclusions:**

Although patients with dementia were less often assessed for risk factors or prescribed medications for secondary prevention of stroke, the control of risk factors did not differ by dementia status. Current findings may reflect appropriate clinical decision‐making for managing people with dementia approaching the end of life.

**Practice impact**

Although we found a lack of equitable general practice care for secondary prevention of stroke between patients with vs without dementia, control of risk factors did not differ by dementia status. Current findings deserve some reflection on decisions about appropriate clinical management of older patients with multiple complex chronic conditions.

## 1 INTRODUCTION

Approximately 10% of people with acute stroke or transient ischaemic attack (TIA) have dementia.^1^ Survivors of stroke with dementia often experience more severe outcomes than survivors without dementia.^2^ Given considerable physical, communication or emotional impairments often experienced by people with dementia and stroke,^2^ clinical management of survivors of stroke with dementia can be challenging. Moreover, given that survival is poorer among patients with stroke having dementia, care needs may differ when compared to patients with stroke not having dementia. For instance, stroke management may be the focus of care, or at other times, patients, carers, family members or clinicians may prioritise management of other comorbidities or end‐of life care.

In Australia, general practitioners (GPs) have an important role in providing ongoing care for people with chronic diseases, such as stroke and dementia. We evaluated any differences, by dementia status, in the management of risk factors in general practices for secondary prevention of stroke.

## 2 METHODS

### 2.1 Study design, setting and data sources

This was a retrospective analysis of adults with acute stroke or TIA from 2014 to 2018 using linked registry and general practice data.^3^ Participants were identified through the Australian Stroke Clinical Registry (AuSCR),^4^ and linked with de‐identified data extracted from electronic medical records of general practices (from 2014 to 2020),^5^ in two metropolitan (Eastern Melbourne and South Eastern Melbourne) and one regional (Gippsland) primary health network in the state of Victoria. The general practice‐sourced data were collected by Outcome Health, via the Population Level Analysis and Reporting (POLAR) platform,^5^ with the permission of the primary health networks as their data custodian. Ethics approval was obtained from the Monash University Human Research Ethics Committee (CF13/1303‐2013000641). The POLAR platform has ethics approval for the collection, transfer and storage of data (RACGP NREEC Protocol ID: 17/008).

### 2.2 Study participants

Participants were eligible if they were discharged home or to inpatient rehabilitation postindex stroke/TIA. Analyses were also restricted to those who survived our observation period of 1 year immediately postacute phase of stroke/TIA (7–18 months postacute event), as patients are usually in the full care of their GPs during this period. Patients discharged to residential aged care were excluded because of the complexity of models of general practice care for residents of aged care facilities in Australia.

To maximise the potential for identifying their regular GP, our cohort was limited to patients who had two or more encounters with the same general practice during the observational period. This approach was necessary as patients may have received primary care for stroke outside of practices covered by the POLAR platform during the observation period. The number of encounters was defined as the sum of days a patient had a record of GP service during the observation period.^6^ We excluded encounters solely for non‐clinical/administrative purpose (e.g. collecting medical certificate), surgical procedures (e.g. biopsy or abscess incision), or for women's health (e.g. pregnancy‐related test and attendance), that are not related to chronic disease management.^7^ To avoid overcounting the frequency of encounters, GP services that occurred within a period of 2 weeks were considered as a single encounter, as they were likely to be related to the same health event.^6^

### 2.3 Definition of patient characteristics

Data collected from general practices comprised diagnoses (e.g. comorbidities), risk factor measures and medications prescribed. Dementia status was determined based on historical recordings of diagnosis codes for dementia (Table S1) or prescription of dementia medications, for example cholinesterase inhibitors (donepezil, galantamine and rivastigmine) and memantine.^8^ Specifically, the study cohort comprised both patients with pre‐ and poststroke/TIA dementia diagnosis (i.e. up to 6 months postindex stroke/TIA). Other comorbidities (e.g. hypertension and diabetes) were defined using diagnoses codes only. The class of medication prescribed was based on the medium level World Health Organization Anatomic and Therapeutic Classification codes.

### 2.4 Evaluation of risk factor management

We evaluated: (i) assessment of cardiometabolic risk factors: blood pressure (BP) from point‐of‐care measurements and serum lipids, blood glucose and urinary proteins from pathology tests; (ii) prescription of prevention medications (BP‐lowering, lipid‐lowering, antithrombotic and glucose‐lowering medications); and (iii) attainment of guideline‐recommended targets for these risk factors (Table S2),^9^, ^10^, ^11^ based on final measurement of each risk factor documented during the observation period.^3^ Data on survival and covariates, for example age, sex, socioeconomic position (based on residential postcode), stroke type and previous history of stroke, were obtained from the AuSCR.

### 2.5 Statistical analysis

Patient characteristics and risk factor management were compared using *χ* ^2^ or Kruskal–Wallis tests. Poisson regression models were used to determine the association between having dementia and the number of risk factors assessed or medication classes prescribed. Logistic regression models were used for analyses of specific risk factors, medication classes or attainment of risk factor targets. All models were adjusted for the number of patient‐GP encounters, all the variables listed in Table 1, and clustering within practices. In sensitivity analyses to minimise bias from imbalance of covariates between groups or any unmeasured contraindication for treatment, we undertook a 1:5 nearest neighbour propensity score matching (calliper = .25 standard deviation) of patients with vs. without dementia. Propensity scores were calculated from a logistic model for dementia regressed on all covariates. Analyses were undertaken using STATA/MP 15.0 (StataCorp, USA, 2017). A two‐sided *p* ≤ .05 was considered statistically significant.

**TABLE 1:** Patient characteristics by dementia status.

	Before matching			After matching		
	No dementia	Dementia	*p*‐Value	No dementia	Dementia	*p*‐Value
Total	3236	140	–	534	138	–
Median age (IQR) at stroke onset (years)	73.2 (63.8, 81.3)	83.4 (78.8, 87.6)	<.001	82.9 (78.7, 86.9)	83.4 (78.8, 87.6)	.34
Median number (IQR) of general practice encounters	6 (4, 8)	7 (5, 11)	.03	7 (5, 9)	7 (4, 9)	.78
Female	1390 (43)	75 (54)	.01	280 (52)	74 (53)	.87
Socioeconomic advantage						
Quintile 1 (least advantaged)	321 (10)	14 (10)	.49	39 (8)	14 (10)	.93
Quintile 2	266 (8)	8 (6)		24 (5)	8 (6)	
Quintile 3	610 (19)	23 (16)		86 (18)	23 (17)	
Quintile 4	809 (25)	32 (23)		120 (25)	32 (23)	
Quintile 5 (most advantaged)	1230 (38)	63 (45)		216 (45)	61 (44)	
Regional location	398 (12)	8 (6)	.02	28 (6)	8 (6)	>.99
Type of stroke			.15			.99
Ischaemic stroke/TIA	2877 (89)	119 (85)		418 (86)	119 (86)	
Intracerebral haemorrhage/Undetermined	359 (11)	21 (15)		116 (14)	19 (14)	
Previous stroke	633 (20)	28 (20)	.90	109 (23)	28 (20)	.59
Discharged to inpatient rehabilitation	1085 (34)	68 (49)	<.001	216 (45)	67 (49)	.40
Comorbidities						
Hypertension	1871 (58)	91 (65)	.09	310 (64)	90 (65)	.78
Diabetes	925 (29)	42 (30)	.72	147 (30)	42 (30)	.98
Atrial fibrillation	668 (21)	42 (30)	.008	143 (30)	42 (30)	.83
Dyslipidaemia	1235 (38)	56 (40)	.66	198 (41)	55 (40)	.84
Cardiovascular disease	643 (20)	39 (28)	.02	123 (25)	39 (28)	.49
Obesity	61 (2)	0 (0)	.18	9 (2)	0 (0)	.22
Kidney disease	260 (8)	26 (19)	<.001	66 (14)	24 (17)	.27
Liver disease	166 (5)	< 5 (<4)	.01	8 (2)	<5 (<4)	.69
Anxiety or depression	809 (25)	58 (41)	<.001	179 (37)	56 (41)	.43
Smoking	82 (3)	< 5 (<4)	.58	6 (1)	<5 (<4)	.56

*Note*: Data are reported as frequencies and proportions, except otherwise stated.

Abbreviations: IQR, interquartile range (Quartile 1, Quartile 3); TIA, transient ischaemic attack.

## 3 RESULTS

### 3.1 Patient characteristics

There were 4476 patients with records of at least one encounter across 383 general practices in the primary health networks investigated (5% with dementia). These included 2487 patients discharged home (4% with dementia), 1414 to inpatient rehabilitation (7% with dementia), 98 to residential aged care (25% with dementia), 352 to other settings (5% with dementia) and 29 with unknown discharge destination (7% with dementia). We excluded 122 patients who died during the observation period. Overall, 3376 patients (75%) were eligible for this study (median age = 73.9 years, 43% females, 22% TIA), including 140 (4%) with dementia (20% TIA). Compared to patients without dementia, those with dementia were older and more often female, discharged to inpatient rehabilitation postindex stroke/TIA event or had more comorbidities (*p* < .05). This imbalance was minimised with propensity score matching (Table 1).

### 3.2 Risk factor management by dementia status

In multivariable analyses, having dementia was associated with having fewer risk factors assessed (incidence rate ratio [IRR] .86, 95% confidence interval [95% CI] .79–.93) or fewer medication classes prescribed (IRR .82, 95% CI .68–.98). Specifically, compared to patients without dementia, those with dementia were significantly less often assessed for BP or serum lipids, or prescribed BP‐lowering, lipid‐lowering or antithrombotic medications (Table 2). No significant difference was observed in attainment of risk factor targets based on dementia status (Table 3). Deprescribing rate from the period within 1 year immediately before stroke/TIA (vs our observation period) was generally greater among patients with dementia (Table S3).

**TABLE 2:** Assessment of risk factors by dementia status (*n* = 3376, including 3236 without dementia and 140 with dementia).

No. of assessments	Blood pressure		Serum lipids		Blood glucose		Kidney function	
	No dementia	Dementia	No dementia	Dementia	No dementia	Dementia	No dementia	Dementia
Median (IQR)	**3 (1, 6)**	**2 (0, 4)**	**1 (0, 1)**	**0 (0, 1)**	1 (0, 1)	1 (0, 1)	0 (0, 1)	1 (0, 2)
0	**615 (19.0)**	**44 (31.4)**	**1453 (44.9)**	**94 (67.1)**	1600 (49.4)	67 (47.9)	1625 (50.2)	68 (48.6)
1–3	**461 (14.3)**	**19 (13.6)**	**1264 (39.1)**	**38 (27.1)**	991 (30.6)	48 (34.3)	810 (25.0)	30 (21.4)
3–4	**1084 (33.5)**	**45 (32.1)**	**512 (15.8)**	**8 (5.7)**	616 (19.0)	22 (15.7)	666 (20.6)	33 (23.6)
≥5	**1076 (33.3)**	**32 (22.9)**	**7 (.2)**	**0 (0)**	29 (.9)	3 (2.1)	135 (4.2)	9 (6.4)

*Note*: *p* ≤ .05 for estimates highlighted in bold.

**TABLE 3:** Odds ratios for the management of risk factors poststroke/TIA according to dementia status.

	Dementia *n* (%)	No dementia *n* (%)	Odds ratio (95% confidence interval)		
			Univariable	Adjusted^a^	1:5 Propensity score matching^a^
	*n* = 140	*n* = 3236	*n* = 3376	*n* = 3376	*n* = 673
Assessment of risk factors					
Blood pressure	**96 (69)**	**2621 (81)**	.**51 (.35, .74)**	.**62 (.43, .92)**	.**57 (.37, .88)**
Serum lipids	**46 (33)**	**1783 (55)**	.**40 (.28, .57)**	.**54 (.37, .79)**	.**54 (.37, .80)**
Blood glucose	73 (52)	1636 (51)	1.07 (.76, 1.50)	1.06 (.76, 1.50)	1.12 (.78, 1.61)
Kidney function	72 (51)	1611 (50)	1.07 (.76, 1.50)	.93 (.62, 1.37)	.89 (.59, 1.34)
Prescription of medications					
BP‐lowering agents	91 (65)	2272 (70)	.79 (.55, 1.12)	.65 (.41, 1.04)	.**58 (.39, .87)**
Lipid‐lowering agents	**86 (61)**	**2342 (72)**	.**61 (.43, .86)**	.71 (.50, 1.02)	.**66 (.45, .98)**
Antithrombotic agents	75 (54)	1816 (56)	.90 (.64, 1.27)	.**61 (.41, .93)**	.**57 (.38, .88)**
Glucose‐lowering agents	26 (19)	556 (17)	1.09 (.71, 1.70)	1.34 (.84, 2.14)	1.15 (.70, 1.89)
Attainment of risk factor targets^b^					
Blood pressure (*n* = 2717)	74/96 (77)	1860/2621 (71)	1.37 (.85, 2.23)	1.54 (.93, 2.55)	1.46 (.87, 2.45)
Serum lipids (*n* = 1554)	17/34 (50)	628/1508 (42)	1.40 (.71, 2.77)	1.18 (.57, 2.46)	1.12 (.52, 2.45)
Blood glucose (*n* = 1489)	47/66 (71)	1098/1388 (79)	.65 (.38, 1.13)	.59 (.33, 1.07)	.58 (.30, 1.12)
Kidney function (*n* = 1722)	24/72 (33)	425/1611 (26)	1.40 (.84, 2.31)	.79 (.40, 1.57)	.78 (.42, 1.45)

*Note*: *p* ≤ .05 for estimates highlighted in bold.

^a^ Adjusted for all variables listed in Table 1.

^b^ Analyses were restricted to patients who were assessed for this risk factor during the observation period and were based on last risk factor measures documented during this period.

## 4 DISCUSSION

We found variation by dementia status in risk factor management for secondary prevention of stroke in Australian general practices. Specifically, patients with dementia were less often assessed for BP and serum lipids, or treated with secondary prevention medications. Primary care‐led integrated management of multimorbidity (e.g. people with stroke and dementia) enhances comprehensive chronic disease management.^12^ It is remarkable that, although patients with dementia were less often assessed for risk factors or prescribed medications for secondary prevention of stroke than those without dementia, attainment of targets did not differ by dementia status. Therefore, our findings may reflect appropriate clinical decision‐making by GPs (as indicated by greater deprescribing rate poststroke among those with dementia) for managing people with dementia approaching the end of life^13^ (Table S4).

The greater prevalence of documented diagnosis of dementia in the most advantaged (vs less disadvantaged) socioeconomic quintiles may be due to the fact that they are more likely to be educated, and thus more likely to seek or have resources to seek, health‐care intervention. The poorer control of blood glucose or urinary protein in patients with dementia vs. without dementia were (albeit non‐significantly due to small cell sizes), may explain the greater prevalence of chronic kidney disease among patients with dementia.

Current findings may be limited by the low, or underestimation of, prevalence of dementia diagnosis in the study cohort. This low prevalence may be primarily due to inaccuracies in dementia diagnosis by GPs,^14^ or reluctance by GPs to diagnose or document diagnosis, potentially due to inherent difficulties in diagnosis which requires a special assessment.^13^, ^15^ Our findings are also limited by the lack of data on functional or cognitive status of patients to ascertain the severity of dementia. Current findings may not reflect the complete picture of management of patients with stroke and dementia, as patients may have received specialist (e.g. neurologists and geriatricians) care during the observation period, which is not captured in POLAR. However, although in Australia, medications for dementia or stroke management are initiated by specialists, GPs often provide the continuation scripts, or initiate treatment via a private script, for ongoing management.

## 5 CONCLUSIONS

We found a lack of equitable care for secondary prevention of stroke between patients with vs. without dementia in general practice. However, control of risk factors did not differ by dementia status. Current findings deserve some reflection on decisions about appropriate clinical management of older patients with complex chronic conditions.

## FUNDING INFORMATION

This study received funding from the Stroke Foundation (Australia) seed grant (ECSeed2019). Dominique Cadilhac received Research Fellowship support from the National Health and Medical Research Council (NHMRC; 1154273), Nadine Andrew (102055) and Monique Kilkenny (10573) from the Heart Foundation. Seana Gall received NHMRC funding (STOPstroke Synergy Grant 1182071).

## CONFLICT OF INTEREST STATEMENT

DAC is the data custodian for AuSCR. DAC, NAL and MFK are members of the AuSCR Management Committee. CP is the data custodian for AURORA. DAC received restricted grants from Boehringer Ingelhiem, Bristol‐Myers, Moleac and Medtronic, and DAC and MFK from Amgen, outside the submitted work.

## Supporting information


Appendix S1

Data S1

## Data Availability

The data that support the findings of this study are available from with the permission of the Primary Health Networks. Restrictions apply to the availability of these data, which were used under license for this study. Data are available from the authors with the permission of the Primary Health Networks.
