# Supplementary material for: Primary care management of stroke in people with dementia: Linked registry and general practice data
Source: Australas J Ageing. 2025 Jul 11;44(3):e70064. doi: 10.1111/ajag.70064 (PMC12247664; doi:10.1111/ajag.70064)
Supplement: Supplementary file 1 — Appendix S1 [file AJAG-44-0-s002.docx]

**Supplementary Material**

**Supplementary Table 1: List of SNOMED-CT-AU diagnosis codes for dementia***

| S/N | SNOMED-CT-AU codes |
| --- | --- |
| 1 | 56267009 |
| 2 | 429998004 |
| 3 | 52448006 |
| 4 | 26929004 |
| 5 | 79341000119107 |
| 6 | 312991009 |
| 7 | 278857002 |
| 8 | 230270009 |
| SNOMED-CT-AU, Systematized Nomenclature of Medicine-Clinical Terms (Australian Version)  * Codes were those documented in the general practice data, and were mapped to International Statistical Classification of Diseases and Related Health Problems, Tenth Revision, Australian Modification (ICD-10-AM) diagnoses using the SnoMAP Starter Web Service (<https://ontoserver.csiro.au/site/our-solutions/snomap-starter/>) | |

**Supplementary Table 2: Target values for risk factor management**

| Risk factor | Target values* |
| --- | --- |
| Blood pressure | Systolic ≤140 mmHg and Diastolic ≤90 mmHg |
| Serum lipids | HDL-Cholesterol >1.0 mmol/L, LDL-Cholesterol <2.0 mmol/L, and Total Cholesterol <4.0 mmol/L; or |
|  | HDL-Cholesterol >1.0 mmol/L, LDL-Cholesterol <2.0 mmol/L, and Triglycerides <2.0 mmol/L |
| Blood glucose | HbA1c ≤7%; or |
|  | Fasting plasma glucose < 7 mmol/L; or |
|  | 2-hour glucose <11.1 mmol/L |
| Kidney function | eGFR <45 mL/min/1.73m^2^; or |
|  | Albumin creatinine ratio creatinine <30 mg/mmol |
| LDL, low-density lipoprotein; HDL, high-density lipoprotein; BMI, body mass index; HbA1c, glycosylated haemoglobin; eGFR, estimated glomerular filtration rate.  *Targets are based on Australian clinical guidelines.^9,10^ | |

**Supplementary Table 3: Percentage reduction in the proportion of patients prescribed classes of prevention medication from the period within 1-year immediately before stroke/TIA *vs*. the observation period (7-18 months post-stroke)**

|  | No Dementia  n=3,236 | Dementia  n=140 |
| --- | --- | --- |
| BP-lowering agents | 12 | 14 |
| Lipid-lowering agents | 11 | 15 |
| Antithrombotic agents | 17 | 26 |
| Glucose-lowering agents | 12 | 5 |

**Supplementary Table 4: Odds ratios for the management of risk factors post-stroke/TIA according to dementia status in patients with multiple general practice visits during the observation period**

|  | Dementia  n (%)  n=147 | No dementia  n (%)  n=3,381 |  | Odds ratio (95% confidence interval) | | |
| --- | --- | --- | --- | --- | --- | --- |
|  |  |  |  | Univariable  n=3,528 | Adjusted ^a^  n=3,528 | 1:5 Propensity score  matching; ^a^ n=719 |
| Assessment of risk factors |  |  |  |  |  |  |
| Blood pressure | **98 (67)** | **2,690 (80)** |  | **0.51 (0.36, 0.73)** | **0.60 (0.41, 0.87)** | **0.58 (0.39, 0.87)** |
| Serum lipids | **48 (33)** | **1,815 (54)** |  | **0.42 (0.29, 0.59)** | **0.54 (0.37, 0.79)** | **0.54 (0.37, 0.78)** |
| Blood glucose | 76 (52) | 1,671 (49) |  | 1.10 (0.79, 1.52) | 1.06 (0.74, 1.50) | 1.06 (0.73, 1.53) |
| Kidney function | 75 (51) | 1,647 (49) |  | 1.10 (0.79, 1.53) | 0.91 (0.62, 1.36) | 0.98 (0.66, 1.31) |
| Prescription of medications |  |  |  |  |  |  |
| BP-lowering agents | 93 (63) | 2,322 (69) |  | 0.79 (0.56, 1.11) | **0.63 (0.40, 0.99)** | **0.62 (0.40, 0.95)** |
| Lipid-lowering agents | **91 (62)** | **2,389 (71)** |  | **0.67 (0.48, 0.95)** | 0.71 (0.53, 1.10) | 0.69 (0.46, 1.03) |
| Antithrombotic agents | 79 (54) | 1,860 (55) |  | 0.95 (0.68, 1.32) | **0.63 (0.42, 0.95)** | **0.66 (0.45, 0.99)** |
| Glucose-lowering agents | 26 (18) | 573 (17) |  | 1.05 (0.68, 1.62) | 1.20 (0.74, 1.95) | 1.14 (0.70, 1.84) |
| Attainment of risk factor targets ^b^ |  |  |  |  |  |  |
| Blood pressure (n=2717) | 76/98 (78) | 1,908/2690 (71) |  | 1.42 (0.87, 2.29) | 1.59 (0.97, 2.60) | 1.49 (0.88, 2.53) |
| Serum lipids (n=1554) | 21/42 (50) | 710/1697 (42) |  | 1.39 (0.75, 2.56) | 1.18 (0.61, 2.27) | 1.45 (0.73, 2.86) |
| Blood glucose (n=1489) | 48/69 (70) | 1,120/1420 (79) |  | 0.61 (0.36, 1.04) | **0.55 (0.31, 0.99)** | 0.58 (0.31, 1.07) |
| Kidney function (n=1722) | 25/75 (33) | 438/1647 (27) |  | 1.38 (0.84, 2.26) | 0.74 (0.38, 1.43) | 0.86 (0.48, 1.53) |
| *p* ≤ 0.05 for estimates highlighted in bold.  ^a^ Adjusted for all variables listed in Table 1; ^b^ Analyses were restricted to patients who were assessed for this risk factor during the observation period, and were based on last risk factor measures documented during this period. | | | | | | |


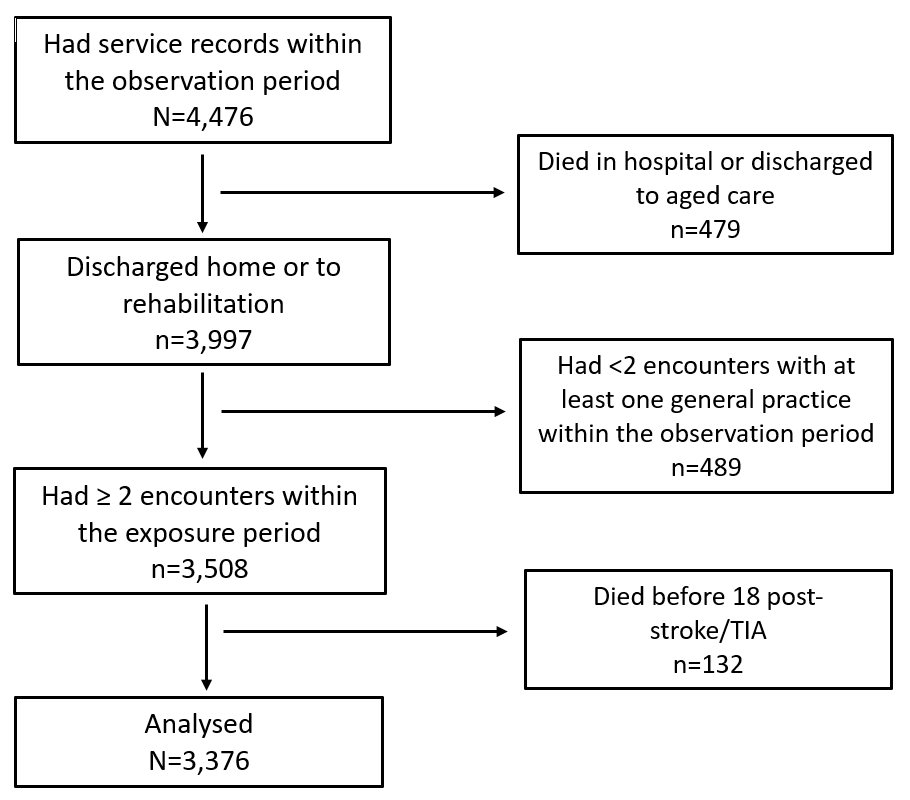


**Supplementary Figure 1:** Flow of patients
