# Supplementary material for: Primary care management of stroke in people with dementia: Linked registry and general practice data
Source: Australas J Ageing. 2025 Jul 11;44(3):e70064. doi: 10.1111/ajag.70064 (PMC12247664; doi:10.1111/ajag.70064)
Supplement: Supplementary file 2 — Data S1 [file AJAG-44-0-s001.docx]

**Supplemental Acknowledgements**

Co-investigators and other contributors to the Australian Stroke ClinicalRegistry (AuSCR)

The following people are acknowledged for their contribution tocollecting hospital data on the patients registered in AuSCR or theirparticipation on various governance committees:

**Steering andManagement Committee:**

Craig Anderson PhD (Royal Prince Alfred Hospital NSW, siteinvestigator, The George Institute for Global Health University of New SouthWales NSW, The George Institute for Global Health at Peking University HealthScience Center China); Geoffrey Donnan MD (Stroke Division, The FloreyInstitute of Neuroscience and Mental Health VIC); Rohan Grimley MBBS (GympieHospital QLD, Nambour General Hospital QLD, site investigator, Sunshine CoastClinical School, University of Queensland QLD); Peter Hand MBBS, MD, FRACP(Royal Melbourne Hospital VIC, site investigator)

**Steering Committee:**

Toni Aslett BASc, GradDipBus (Stroke Foundation); Julie Bernhardt PhD(The Florey Institute of Neuroscience and Mental Health VIC); Paul Bew MPhty(The Prince Charles Hospital QLD); Christopher Bladin MD, MBBS, FRACP (Box HillHospital VIC, site investigator); Greg Cadigan BN (Queensland State-wide StrokeClinical Network QLD); Helen Castley MBBS (Royal Hobart Hospital Tasmania, siteinvestigator); Andrew Evans MBBS (Hons), FRACP (Westmead Hospital NSW); SusanHillier PhD (University of South Australia, SA); Erin Lalor PhD (StrokeFoundation VIC); Andrew Lee MBBS FRACP (Flinders Medical Centre, SouthAustralia); Richard Lindley PhD (The George Institute for Global Health NSW);Mark Mackay MBBS, FRACP (Royal Children’s Hospital VIC, site investigator);Sandra Martyn (Health Statistics Centre Queensland Health QLD); John McNeil PhD(Monash University VIC); Sandy Middleton PhD (Nursing Research Institute, StVincent’s Health Australia NSW, Australian Catholic University NSW); MichaelPollack MBBS, FAFRM (RACP), FACRM, FFPM (ANZCA), MMedSci (Clin Epi) (HunterStroke Service NSW); Mark Simcocks BSc (VIC, Consumer Representative); FrancesSimmonds MSc(Med), (Australasian Rehabilitation Outcomes Centre NSW); AndrewWesseldine MBBS, FRACP (St John of God Healthcare; Department of Health WA)

**ManagementCommittee:**

Helen Dewey PhD (Austin Hospital VIC, Box Hill Hospital VIC, siteinvestigator, Eastern Health Clinical School, Monash University VIC); StevenFaux FAFRM (RACP) (St Vincent’s Health Australia NSW); Kelvin Hill BAppSci(Stroke Foundation VIC); Christopher Levi PhD (Acute Stroke Services, JohnHunter Hospital NSW, site investigator); Christopher Price BSocW BSc (NationalStroke Foundation)

**Site Investigators:**

Lauren Arthurson BSpPath, MHlthServMt (Echuca Regional Health VIC);Pradeep Bambery MD, FRCP(G), FRACP (Bundaberg Hospital QLD); Tim Bates MBBS,FRACP (Swan District Hospital WA); Carolyn Beltrame RN (Div1) (Latrobe RegionalHospital VIC); David Blacker MBBS, FRACP (Sir Charles Gairdner Hospital WA);Ernie Butler MBBS FRACP (Peninsula Health VIC); Sean Butler FIMLS, BM Hons,MRCP(UK), FRACP (Prince Charles Hospital QLD); Chris Charnley MBBS (WarrnamboolBase Hospital VIC); Ben Clissold MBBS FRACP (University Hospital Geelong VIC);Jo Cotterell BPhysio (Mildura Base Hospital VIC); Douglas Crompton MA, PhD,MBBS, FRACP (Northern Hospital VIC); Vanessa Crosby Dip Physio (Albury-WodongaHealth VIC); Carolyn De Wytt MRCP (UK), MB BCH DUBL, FRACP (Greenslopes PrivateHospital QLD); David Douglas MBBS, M Admin, FRACGP, FAFRM (RACP) (IpswichHospital QLD); Martin Dunlop MBBS, FACRM (Cairns Base Hospital QLD); PaulaEaston BPhty (Hons) (Mackay Hospital QLD); Sharan Ermel RN (Div1) (BendigoHealth VIC); Nisal Gange MBBS, AMC CERT (Toowoomba Hospital QLD); RichardGeraghty MBBS, FRACP (Redcliffe Hospital QLD); Melissa Gill BAppSc (SpPath)(Armidale Hospital, NSW); Kushantha Gunarathne MBBS, MD, MMed, FRCP, FRACP(Bairnsdale Regional Health Service VIC); Graham Hall MBBS, FRACP (PrincessAlexandra Hospital QLD); Geoffrey Herkes MBBS, PhD, FRACP (Royal North ShoreHospital NSW); Jonelle Hill-Uebergang BNursing, GradDip Advanced ClinicalNursing, Advanced Dip Management (Northeast Health Wangaratta VIC); Karen HinesBHIM (Caboolture Hospital QLD); Francis Hishon RN (Redland Hospital QLD); JamesHughes BMed, FRACP (Tamworth Hospital NSW); Joel Iedema MBBS, FRACP (RedlandHospital QLD); Martin Jude MBBS, FRACP (Wagga Wagga Hospital NSW); ThomasKraemer Approbation als Arzt, STATE EXAM MED MUNSTER, FRACP (Ballarat HealthServices VIC); Paul Laird MBBS, FRACP (Rockhampton Hospital QLD); Henry Ma MBBSFRACP  (Monash Medical Centre VIC);Johanna Madden BPhysio (Goulburn Valley Health VIC); Graham Mahaffey RN (HerveyBay Hospital QLD); Krishna Mandaleson MBBS, FRACP, FRCP (Central GippslandHealth Service VIC); Suzana Milosevic MD, FRACP, AMC CERT (Logan Hospital QLD);Peter O’Brien MBBS, DIP RANZCOG, FRACMA, FACRRM (Warrnambool Hospital VIC);Trisha Oxley RN/RM, MANP (Critical Care) (Swan Hill District Health VIC);Michaela Plante RN (Div 1) (Rockhampton Hospital QLD); Stephen Read MBBS, PhD,FRACP (Royal Brisbane and Women’s Hospital QLD); Dane Robinson B Occ Thy(Prince Charles Hospital QLD); Juan Rois-Gnecco Medico Cirujano Javeriana,FAFRM (Ipswich Hospital QLD); David Rosaia BHlthSc, GradDipHlthSc (BendigoHealth VIC); Kristen Rowe BNurs, Cert NeuroSci Nurs (Austin Health VIC); FionaRyan BAppSc (SpPath), MHlthSc (Orange Hospital and Bathurst Hospitals NSW);Arman Sabet MD, FRACP, BSc (Gold Coast Hospital and Robina Hospital QLD); NoelSaines MBBS, FRACP (The Wesley Hospital QLD); Eva Salud MD, AMC CERT (GympieHospital QLD); Amanda Siller MBBS, FRACP (Queen Elizabeth II Jubilee HospitalQLD); Christopher Staples MD (Mater Adults QLD); Amanda Styles RN (Div 1)(Armidale Hospital NSW); Vincent Thijs MD FRACP PhD (Austin Hospital VIC);Dinesh Tryambake MBBS FRCP (Launceston General Hospital TAS); JudithWalloscheck MBA (Bendigo Health VIC); Richard White MD, FRCP, FRACP (TownsvilleHospital QLD); Tissa Wijeratne (Sunshine Hospital - Western Health VIC); AndrewWong MBBS, PhD (Royal Brisbane and Women’s Hospital QLD); Lillian Wong MBBSFRACP (Logan Hospital QLD); Jorge Zavala MD FRACP (Alfred Hospital VIC)

**Staff at The FloreyInstitute of Neuroscience and Mental Health VIC**

Robin Armstrong, Leonid Churilov, Alison Dias, Kelly Drennan, AdeleGibbs, Brenda Grabsch, Elysia Greenhill, Jen Holland, Charlotte Krenus, FrancisKung, Joyce Lim, Karen Moss, Kate Paice, Enna Salama, Sam Shehata, SabrinaSmall, Renee Stojanovic, Steven Street, Emma Tod, Kasey Wallis, Julia Watt
